# Influence of Solid Solution Treatment on Microstructure and Mechanical Properties of 20CrNiMo/Incoloy 825 Composite Materials

**DOI:** 10.3390/ma17225588

**Published:** 2024-11-15

**Authors:** Jie Liu, Qiang Li, Hailian Gui, Peng Zhang, Sha Li, Chen Zhang, Hao Liu, Chunlei Shen, Pengyue Zhang

**Affiliations:** 1 School of Materials Science and Engineering, Taiyuan University of Science and Technology, Taiyuan 030024, China; b202114310013@stu.tyust.edu.cn (J.L.); 2022107@tyust.edu.cn (S.L.); b202314110024@stu.tyust.edu.cn (C.Z.); b202214110015@stu.tyust.edu.cn (H.L.); 2 China School of Intelligent Engineering, Jinzhong College of Information, Jinzhong 030800, China; 3 Department of Mechanical Engineering, Taiyuan University of Science and Technology, Taiyuan 030024, China; 4 Shanxi Provincial Key Laboratory of Metallurgical Device Design Theory and Technology, Taiyuan 030024, China; s202247211229@stu.tyust.edu.cn; 5 Jiangsu Aijisi Haizhu Machinery Co., Ltd., Huai’an 215000, China; 18935453205@163.com; 6 Shanxi Ganghe Li New Material Technology Co., Ltd., Taiyuan 030024, China; 18234419288@163.com

**Keywords:** 20CrNiMo/Incoloy 825 composite material, solid solution treatment, microstructure, tensile, shear

## Abstract

The utilization of 20CrNiMo/Incoloy 825 composite materials as high-pressure pipe manifold steel can not only improve the strength and hardness of the steel, but also improve its corrosion resistance. However, research on the heat treatment of 20CrNiMo/Incoloy 825 composite materials is still scarce. Thus, the aim of this study was to investigate the influence of solid solution treatment on the microstructure and properties of 20CrNiMo/Incoloy 825 composite materials. Firstly, the composite materials were subjected to solid solution treatment at temperatures ranging from 850 to 1100 °C with varied holding times of 1 h, 4 h, and 6 h. Microstructural analysis revealed that the solid solution treatment temperature had a more pronounced effect than the treatment time on the interface decarburization layer, carburization layer, and grain size. It was observed that the carburized layer thickness decreased while the decarburized layer thickness increased with an increase in the solid solution treatment temperature, oil cooling was found to enhance the hardness of the base layer of the composite materials, and the size of the original austenite grains of 20CrNiMo steel and Incoloy 825 increased with an increase in the solid solution treatment temperature. Secondly, the tensile properties, microhardness, and fracture morphology were evaluated after the composite materials underwent solid solution treatment at temperatures between 950 °C and 1100 °C for 1 h. The results indicated that increasing the solution temperature initially led to an increase in tensile strength and elongation after fracture, followed by a decrease; furthermore, the hardness of Incoloy 825 exhibited a declining trend, while the hardness of 20CrNiMo first decreased then increased. Thirdly, the shear properties and interfacial element diffusion of the composite materials were analyzed following solid solution treatment in a temperature range of 950 °C to 1100 °C for 1 h. The findings demonstrated that higher solid solution treatment temperatures induced full diffusion of Cr, Ni, and Fe atoms at the interface and softened the matrix, leading to an increase in the thickness of the diffusion layer and toughening of the composite interface. Therefore, the shear strength increased with an increase in the solid solution treatment temperature. Finally, the optimal solid solution treatment process for 20CrNiMo/Incoloy 825 composite materials was determined to be 1050 °C/1 h oil cooling, following which the composite materials had good comprehensive mechanical properties.

## 1. Introduction

High-pressure manifold systems consist of movable elbows, tees, straight pipes, fittings, and other components, which are mainly used for collecting and transporting fracturing fluids discharged from fracturing pumps and high-pressure fluids returned from subsurface formations [1,2]. At present, the materials commonly utilized for high-pressure pipe manifolds include CrMo series steel and CrNiMo series steel. Specifically, CrMo and CrNiMo series steel are primarily utilized for high-pressure pipe manifolds with pressures below and exceeding 70 MPa, respectively. At present, there is considerable scholarly interest in the scrutiny of 15CrNiMo steel. Professor Guo Dengming has conducted diverse heat treatments for 15CrNiMo steel. Guo Dengming et al. conducted a comparative analysis of the microstructure, hardness, and wear resistance of 15CrNiMo steel and revealed that its working surface can achieve a mixed microstructure of lower bainite and tempered martensite after carburizing, isothermal quenching, and low-temperature tempering processes, which improve the overall mechanical properties of the material [1]. Yu Hongjun et al. delved into the influence of the heat treatment process on the microstructure and properties of 15CrNiMo steel, highlighting the enhanced properties exhibited by steel subjected to direct tempering [3]. Ji Ling et al. compared the impact of different rolling downline temperatures on the microhardness, microstructures, and microstructural properties of 15CrNiMo steel, revealing that elevating the rolling downline temperature effectively augments the ferrite structure in 15CrNiMo steel, resulting in reduced steel hardness and hydrogen content [4]. Nevertheless, high-pressure pipe manifolds made of 15CrNiMo steel face challenges such as high-speed erosion from solid particle-laden fluids during service and the corrosive effects of fracturing fluids or high-pressure fluids. Consequently, these manifolds are susceptible to fatigue cracks and corrosive leakage, potentially leading to rupture and perforation, causing equipment damage, and posing a risk to personnel safety. To mitigate these concerns, PANDA investigated the tribo-corrosion inhibition of a 15CrNiMo steel pipe carrying hydraulic fracturing fluid. They found that the chemical adsorption of the corrosion inhibitor molecules on the steel surface enhances the corrosion resistance of the steel [5]. However, the improvement provided by these inhibitors is limited. To further enhance corrosion resistance, Incoloy 825 was selected as the composite material. To enhance the strength and hardness of the steel utilized in high-pressure pipe manifolds, 20CrNiMo steel was selected in the present work as the base material instead of 15CrNiMo steel, and the rolling process of the 20CrNiMo/Incoloy 825 composite material was studied.

The rolling process—as the most extensively utilized bimetallic composite method—facilitates the metallurgical bonding of dissimilar metals through the application of substantial rolling forces and thermal diffusion of the constituent elements [6,7]. Moreover, the interfacial bonding properties of the composite materials are observed to improve with an increase in the rolling pressure. Rui Cao et al. conducted a study in which they prepared 2Cr13/316L multilayer composite materials via hot rolling, incorporating a cyclic heating step, and reported that the interfacial bonding properties positively correlated with the increase in rolling pressure [8]. Similarly, Juan Li employed distinct processes involving the hot rolling and heat treatment of NM500/Q345/NM500 composite plates and noted that enhanced rolling pressure facilitated the promotion of metallurgical bonding between NM500 and Q345 steels [9]. However, it is essential to note that rolled composite materials may be prone to several defects, including non-uniform processing stresses, work hardening, and inadequate matching of the microstructure distribution and strength–ductility [10,11]. All these defects affect the properties of the composite material, and this study proposes heat treatment for the rolled composite materials in order to address this phenomenon. There are also several studies on the heat treatment of composite materials. Jian Zhang et al. examined the influence of heat treatment temperature on the tensile fracture behavior of AZ31/6061 exploded composites, elucidating the temperature’s effect on the interfacial strengthening mechanism and fracture behavior of Mg/Al composites during the fracture process [12]. Qiangqiang Zhu et al. conducted research on the solution aging treatment of hot-rolled thin Ti-6Al-4V alloy plates and identified that the most optimal strength–plasticity match could be achieved through treatment at 930 °C for 60 min or 960 °C for 30 min [13]. Furthermore, Nan Zhang et al. investigated the impact of annealing on the interfacial structure evolution and mechanical properties of AZ31B/AA6061 composites, observing an initial increase in tensile strength followed by a sharp decrease, with a simultaneous significant increase in elongation as the annealing temperature was raised [14]. Despite the extensive research on the effect of heat treatment on composite materials, limited attention has been dedicated to the application of heat treatment to stainless steel composite materials. Furthermore, there is a notable dearth of research concerning the heat treatment of 20CrNiMo/Incoloy 825 composite materials.

Based on this, the effect of solid solution treatment on the microstructure and mechanical properties of 20CrNiMo/Incoloy 825 composite materials was investigated in this study. Firstly, different solid solution treatment processes involving 20CrNiMo/Incoloy 825 composite materials were carried out to determine the solid solution treatment time and cooling mode through microstructure analysis. Secondly, the tensile properties, microhardness, and fracture morphology of the composite materials at different solid solution treatment temperatures were analyzed, and the solid solution treatment process was initially determined based on the tensile strength, hardness, and fracture morphology. Finally, the shear properties, microhardness, and interfacial element diffusion of the composite materials at different solid solution treatment temperatures were analyzed, and the optimum solid solution treatment process for 20CrNiMo/Incoloy 825 composite materials was determined.

## 2. Experimental Materials and Procedures

20CrNiMo steel was used as the base material, and Incoloy 825 was used as the compound layer. The compositions of the two materials are shown in Table 1. The 20CrNiMo and Incoloy 825 materials were cut into sizes of 110 × 100 × 20 mm and 110 × 100 × 10 mm, respectively, using electrical discharge machining. A symmetrical billet was formed through vacuum electron beam welding and was then heated to 1150 °C and held for 1 h. The billet was subsequently rolled on a Φ450 × 450 mm two-roll reversible mill, with a total reduction of 85% in 5 passes. The 20CrNiMo/Incoloy 825 composite materials were successfully prepared.

**Figure 1:**
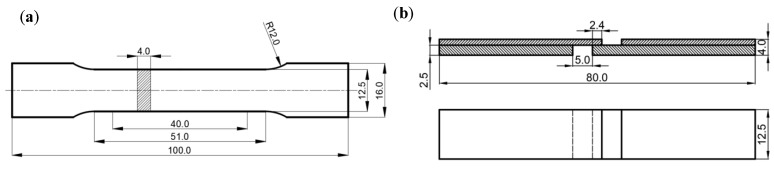
Specimen size: (**a**) tensile sample; (**b**) shear sample.

**Figure 2:**
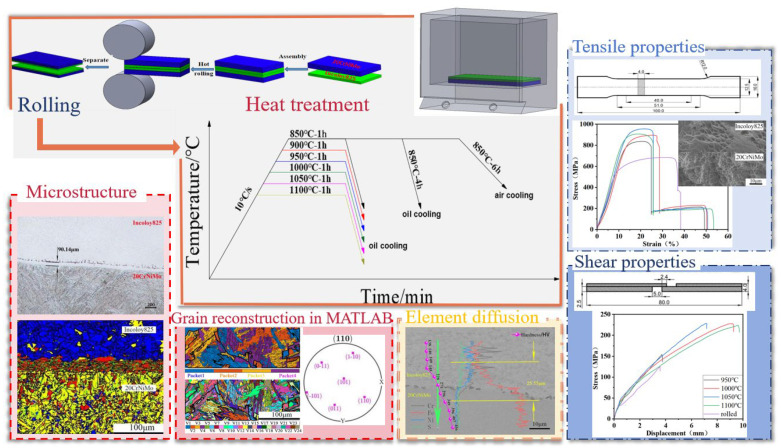
Flow diagram of the experiment.

The 20CrNiMo/Incoloy 825 composite materials underwent solid solution treatment with various temperatures and times, including 850 °C/1 h oil cooling, 850 °C/4 h oil cooling, 850 °C/6 h oil cooling, 850 °C/6 h air cooling, and 950–1100 °C/1 h oil cooling, and the resulting microstructure was analyzed using an optical microscope (OM), electron backscattering diffraction (EBSD), and the MATLAB R2024a software. Subsequently, the composite materials were subjected to 950–1100 °C/1 h oil cooling solid solution treatment. Tensile and shear specimens were then prepared according to the dimensions depicted in Figure 1. These specimens were used for conducting tensile and shear tests employing a WDW-200E universal tensile testing machine. In the tensile tests, the tensile displacement rate was set to 1 mm/min, and three tensile samples were prepared under the same solid solution conditions, with the tensile strength taken as the average of three experiments. For the shear tests, the tensile speed was set to 0.5 mm/min, and three shear samples were prepared under the same solid solution conditions, with the shear strength taken as the average of three experiments. After the tensile and shear tests, the microstructure was characterized using a scanning electron microscope (SEM) and an energy-dispersive spectroscope (EDS). Finally, hardness tests were conducted on the samples along a direction perpendicular to the interface before and after solution treatment using a digital microhardness tester. A load of 100 g was applied for a duration of 10 s, and the hardness value at each point was taken as the average of three measurements. The experimental procedure is outlined in Figure 2.

## 3. Results and Analysis

### 3.1. Effect of Solid Solution Treatment on Microstructure

**Figure 3:**
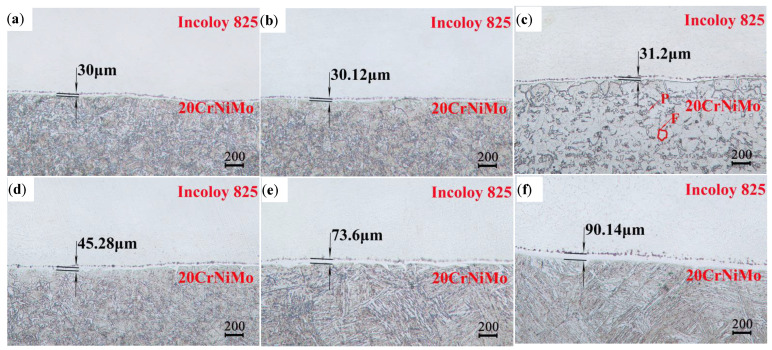
Effect of solid solution treatment on the interface of 20CrNiMo/Incoloy 825 composite materials: (**a**) 850 °C/1 h oil cooling; (**b**) 850 °C/4 h oil cooling; (**c**) 850 °C/6 h air cooling; (**d**) 950 °C/1 h oil cooling; (**e**) 1050 °C/1 h oil cooling; (**f**) 1100 °C/1 h oil cooling.

The effects of different solid solution treatment times on the interface characteristics of 20CrNiMo/Incoloy 825 composite materials are depicted in Figure 3a–c. These figures demonstrate the presence of carburized and decarburized layers. It is noteworthy that the thickness of the decarburized layer slightly increased from 30 μm to 31.2 μm with an extension in the solid solution treatment time, and the change was not obvious. The samples in Figure 3a,b underwent oil cooling subsequent to the solid solution treatment, resulting in the formation of martensitic microstructures in the 20CrNiMo steel with residual austenite. In contrast, the cooling method shown in Figure 3c after solid solution treatment is air cooling, which did not reach the critical speed of martensite formation due to the slow cooling rate, resulting in the final microstructure of ferrite and a small granular pearlitic microstructure. The martensitic microstructure had higher hardness than the ferrite + pearlitic microstructure, which means that the hardness of 20CrNiMo after air cooling was lower than that after oil cooling. The oil cooling solid solution treatment process was selected in order to ensure the hardness of the base layer of the composite material after solid solution treatment.

Figure 3a,d–f depict the influence of varying solid solution treatment temperatures on the interface of 20CrNiMo/Incoloy 825 composite materials. As can be seen from the figure, the thickness of the carburized layer decreased gradually with the increase in temperature, and the carburized layer showed a discontinuous state and was close to disappearing when it reached 1100 °C. Specifically, at solid solution treatment temperatures of 850 °C, 950 °C, 1050 °C, and 1100 °C, the thickness of the decarburized layer measured 30 μm, 45.28 μm, 73.6 μm, and 90.14 μm, respectively. Additionally, the decarburized layer thickened progressively with the increase in solid solution treatment temperature, indicating that the temperature exerted a more significant influence on the decarburized layer than the treatment time. Ding Yun et al. [15] found that the Cr element on the side of the Incoloy 825 diffused to the side of the 20CrNiMo steel, forming a large amount of Cr_23_C_6_ distributed along the grain boundary at the transition layer on the 20CrNiMo side, and Cr_23_C_6_ was formed under the conditions of long-term thermal insulation [16]. Cr_23_C_6_ would lead to the composite materials being prone to intergranular fracture, so the holding time should be minimized when solid solution treatment is carried out.

Moreover, Liu et al. [8,17] asserted that the development of a carburizing layer can lead to the generation of intergranular tunneling cracks, resulting in reduced resistance to corrosion and fatigue. Notably, when subjected to a quenching temperature of 1100 °C, the thickness of the carburized layer was significantly reduced, almost to the point of disappearance. This indicates that high-temperature, short-term solid solution treatment can modify the intergranular corrosion tendency and fracture patterns. Therefore, this research advocates for high-temperature, short-term solid solution treatment of 950 °C/1 h, 1000 °C/1 h, 1050 °C/1 h, and 1100 °C/1 h.

**Figure 4:**
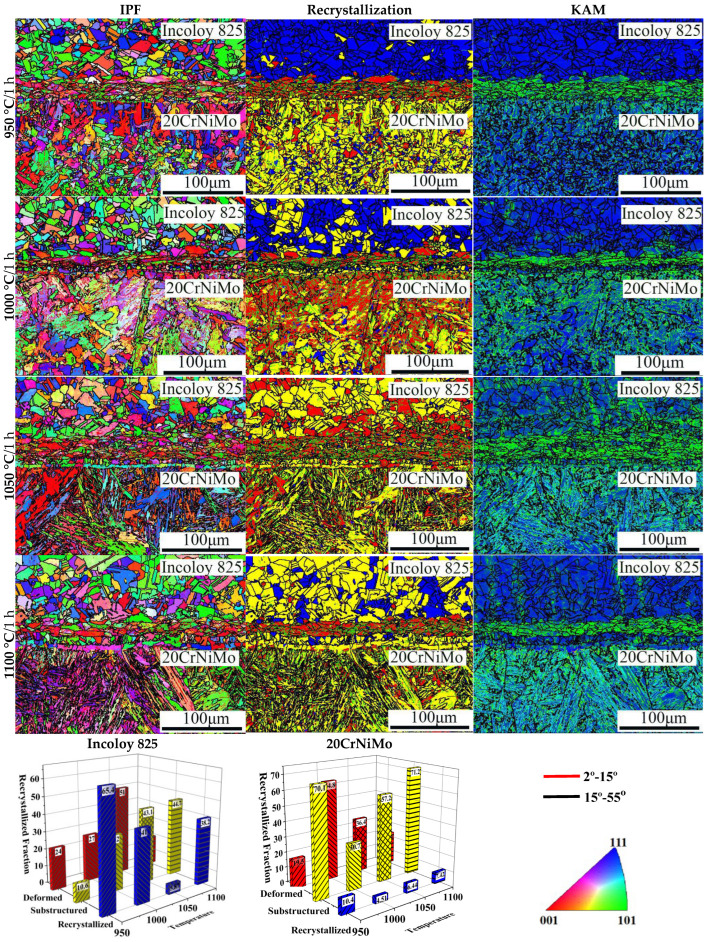
EBSD plots of 20CrNiMo/Incoloy 825 composite materials under different conditions.

Based on the foregoing analysis, it is apparent that the solid solution treatment temperature significantly impacts the bonding interface of 20CrNiMo/Incoloy 825 composite materials. The EBSD technique was employed to comprehensively investigate the impact of various solid solution treatment temperatures on the microstructure of 20CrNiMo/Incoloy 825 composite materials. The outcomes are presented in Figure 4. In the figure, the red line represents orientation difference angles between 2° and 15°, while the black line denotes orientation difference angles exceeding 15°. Within the KAM diagram, blue signifies low dislocation density, and green signifies high dislocation density.

Figure 4 reveals that the microstructure of 20CrNiMo steel consisted of martensite and a small amount of residual austenite. As the solid solution temperature increased, the original austenite grain size of 20CrNiMo steel was enlarged, accompanied by a rise in dislocation density. Furthermore, the areas with high dislocation density were mainly located at the boundary of the plate martensite bundle, forming dislocation cells, and there were a large number of entangled dislocations on the cell wall, while the internal dislocation density of the plate martensite bundle was low [18]. Considering the overall distribution of dislocations, a high density of parallel deformation bands hindered dislocation movement and played a role in dislocation strengthening. Near the interface, 20CrNiMo steel exhibited a minor decarburization tendency, resulting in the hardness of 20CrNiMo steel near the interface being lower than that of the matrix.

Incoloy 825 exhibited an austenitic structure, as depicted in Figure 4. At a solid solution temperature of 950 °C, static recrystallization of the Incoloy 825 transpired, resulting in a grain size of 18.068 μm. Subsequently, when the solid solution temperature was increased to 1000 °C, the grain size measured 19.901 μm, with minimal changes observed in grain size. The recrystallization process of the Incoloy 825 concluded upon reaching a solid solution temperature of 1050 °C, yielding a grain size of 23.113 μm. Further elevation of the solid solution temperature to 1100 °C led to a considerable increase in grain size, which measured 27.336 μm. Consequently, the austenite grain size of the Incoloy 825 layer increased with the solid solution temperature. Moreover, the Incoloy 825 microstructure near the interface was stretched, and a large number of small angle grain boundaries appeared in the grains, resulting in larger dislocation density. This indicates that there was more stored distortion energy near the interface on the Incoloy 825 side, which means that this location had high hardness and poor ductility.

**Figure 5:**
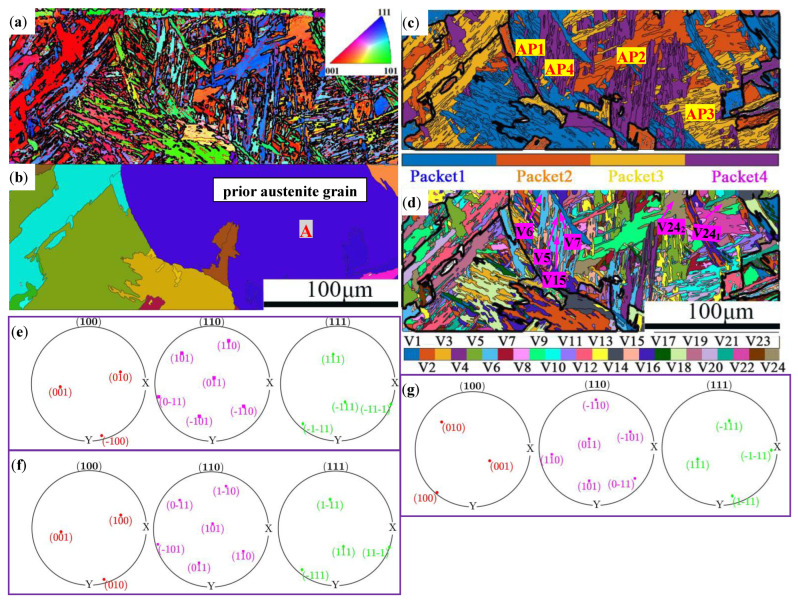
Map of 20CrNiMo steel after lath martensite transformation following solid solution treatment at 1050 °C/1 h: (**a**) IPF map; (**b**) original austenite grain distribution map; (**c**) martensite packet distribution map; (**d**) martensite variants distribution map; (**e**) the {001}, {011}, and {111} pole figures of martensite variant V24_1_; (**f**) the {001}, {011}, and {111} pole figures of martensite variant V24_2_; (**g**) the {001}, {011}, and {111} pole figures of martensite variant V6.

The microstructure of the martensite in 20CrNiMo steel was intricate, rendering Figure 4 inadequate in capturing the alteration of the original austenite grain size. The martensite variant orientation and the reconstruction of the austenite grain from the parent phase of 20CrNiMo steel plates were carried out to comprehensively analyze the original austenite grain size and the martensitic microstructure of 20CrNiMo steel. These processes were computed and scrutinized using MATLAB software under the solid solution treatment conditions of 1050 °C/1 h based on the orientation matrix of the Kurdjumov–Sachs (K-S) relationship. Initially, the martensite variant orientation (24 variant orientations, V1-V24) was computed. Subsequently, the inverse calculation of the original austenite orientation was performed using the relationship between the martensite orientation and phase transition orientation. This analytical approach yielded the findings illustrated in Figure 5, wherein Figure 5a exhibits the martensite inverse pole figure (IPF), Figure 5b portrays the reconstructed original austenite figure, and Figure 5c visualizes the distribution of the martensite packet. Furthermore, Figure 5d displays the distribution of the martensite variants, and Figure 5e–g depict the pole figures of {100}, {110}, and {111} for martensite variants V6, V24_1_, and V24_2_, respectively.

In Figure 5, it can be seen that in lath martensite, an original austenite grain contains multiple martensite packets, each of which can be further divided into several martensitic blocks, and a block consists of several laths [19,20]. For instance, original austenitic grain A contains several packets, such as Packet1 (AP1), Packet2 (AP2), Packet3 (AP3), and Packet4 (AP4), as depicted in Figure 5b,c. It is found that the martensite laths within each packet share the same habit planes, and the martensite laths within each block have the same or a similar crystallographic orientation [21].

A total of 24 martensite variants were calculated in this study using the MATLAB software, as illustrated in Figure 5d. Different colors represent different types of martensite variants, with the 24 variants labeled as V1 to V24. It can be observed that multiple oriented variants existed after the quenching of the original austenite. Furthermore, the orientation characteristics of the martensite generated from different austenites varied. As seen in Figure 5c,d, a martensite packet is composed of several martensite variants, such as AP4 consisting of the martensite variants V5, V6, V7, and V15. The pole figures of {100}, {110}, and {111} were calculated for the variants V24_1_, V24_2_, and V6, shown in Figure 5e–g, respectively. It is evident from the figure that the pole positions of {100}, {110}, and {111} for variants V6 and V24_1_ differed. Conversely, the pole positions of {100}, {110}, and {111} for variants V24_1_ and V24_2_ were identical, indicating that the crystal orientations of variants V6 and V24_1_ were different, while the crystal orientations of variants V24_1_ and V24_2_ were the same. This implies that when the martensite variants were of different types, their crystal orientations were different; conversely, when the martensite variants were of the same type, their crystal orientations were also the same. Each martensite lath represents a single crystal and is the smallest unit of martensite, with each having a fixed orientation. According to the K-S orientation relationship, the orientation difference between variants was either less than 21° or greater than 47°. The orientation difference angle between two neighboring variants falling within the range of 21–47° indicates that the two variants originated from different austenite grains of the parent phase, and the interface between these two variants can be considered as the grain boundary of the parent phase austenite [22]. Based on this, the original austenite grains in martensite were reconstructed, as illustrated in Figure 5b.

**Figure 6:**
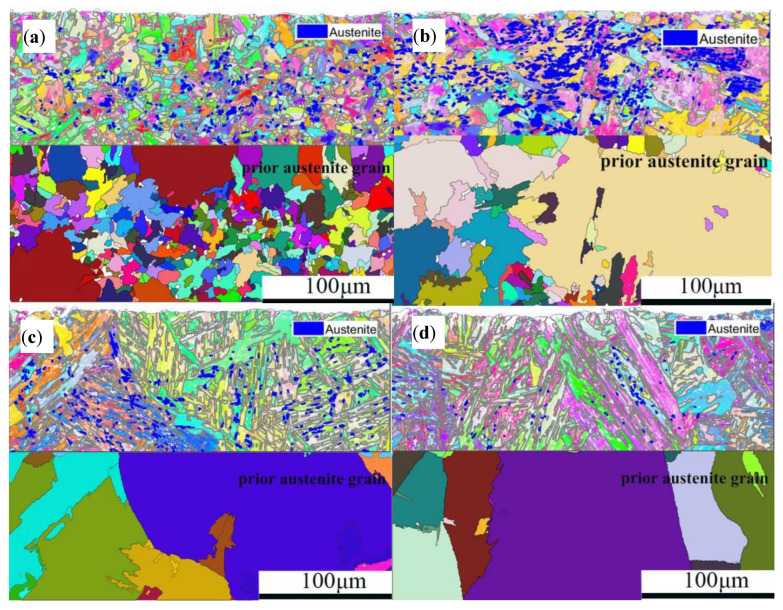
Distribution maps of original austenite and residual austenite in steel 20CrNiMo under different solid solution treatment conditions: (**a**) 950 °C/1 h; (**b**) 1000 °C/1 h; (**c**) 1050 °C/1 h; (**d**) 1100 °C/1 h.

The analysis of the original austenite and residual austenite of 20CrNiMo steel under solid solution treatment conditions ranging from 950 °C/1 h to 1100 °C/1 h was conducted based on the aforementioned principle using MATLAB software, as depicted in Figure 6. The figure’s upper section displays residual austenite distribution, while the lower section illustrates the distribution of original austenite. As illustrated in Figure 6, the blue dots in the figure represent the distribution of residual austenite. the residual austenite increased first and then decreased as the solid solution treatment temperature increased, reaching its maximum at 1000 °C/1 h. The original austenite grain size in 20CrNiMo steel augmented with the solid solution treatment temperature. At a solid solution treatment temperature of 950 °C, the average grain size was small; however, there was considerable variation in grain size, with an uneven distribution. Moreover, the grains in proximity to the composite interface exhibited larger dimensions, whereas those further from the interface were smaller.

### 3.2. Effect of Solid Solution Treatment on Tensile Mechanical Properties

**Figure 7:**
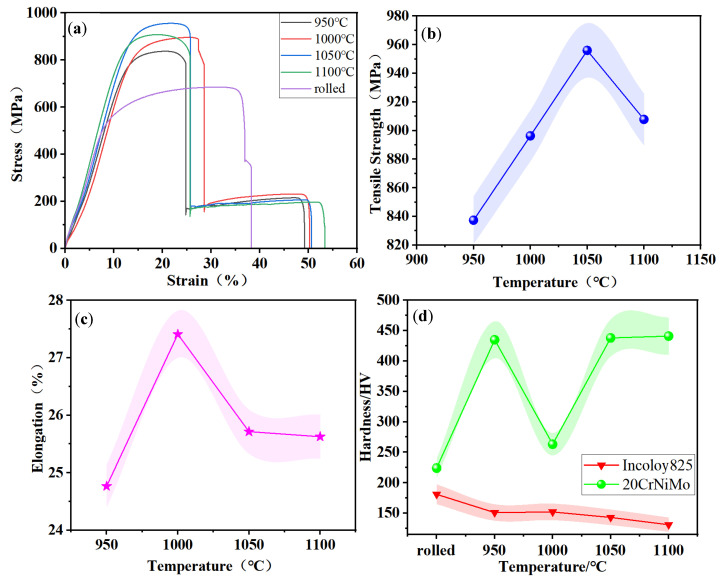
Mechanical properties of 20CrNiMo/Incoloy 825 composite materials in different solid solution treatment processes: (**a**) the tensile stress–strain curve; (**b**) the tensile strength–temperature curve; (**c**) the elongation–temperature curve; (**d**) the hardness–temperature curve.

Tensile and hardness tests were carried out in order to better examine the effect of solid solution treatment temperature on the strength, plasticity, and hardness of the composite materials, and the experimental results are shown in Figure 7. In Figure 7a, it can be seen that the tensile strength of the specimen subjected to rolling without solid solution treatment was 685 MPa. In contrast, the tensile strength of the specimen treated with solid solution treatment exhibited varying degrees of enhancement in tensile strength. Figure 7b illustrates that tensile strength initially increased and then decreased as the solid solution treatment temperature rose. This can be attributed to two factors: firstly, the second phase dissolved into the matrix to form a solid solution with a strengthening effect; secondly, the dislocation density increased with the increase in solid solution treatment temperature, resulting in dislocation strengthening. Nevertheless, increasing the solid solution treatment temperature resulted in a larger grain size of martensite and a wider gap between the lath bundles, leading to reduced tensile strength [23]. The highest tensile strength of 956.007 MPa was obtained at a solid solution treatment temperature of 1050 °C.

As shown in Figure 7c, the impact of solid solution treatment temperature on elongation after fracture was characterized by an initial increase followed by a subsequent decline in the composite materials. During the tensile specimen fracture process, 20CrNiMo steel exhibited initial fracture, followed by Incoloy 825, indicating the superior plasticity of Incoloy 825 compared to 20CrNiMo steel. This suggests that the plasticity of 20CrNiMo/Incoloy 825 composite materials was predominantly influenced by the plasticity of 20CrNiMo steel. Additionally, Figure 6 demonstrates that the residual austenite content in 20CrNiMo steel followed a similar pattern of an initial increase and subsequent decrease with the solid solution treatment temperature. This trend is consistent with the composite’s elongation; the variation in elongation was mainly affected by residual austenite. According to the literature [24], higher temperatures cause widening of the decarburized layer of the substrate and an increase in the number of brittle phases at the side interface of the composite material, which reduces the ability of the composite material to deform uniformly and plastically during stretching and also leads to decreases in the elongation at 1050 °C and 1100 °C.

In Figure 7d, the impact of solid solution treatment temperature on hardness is evident. The trend indicates a decrease in the hardness of Incoloy 825 as the solid solution treatment temperature rises. Moreover, the hardness of 20CrNiMo initially decreases with the rise in solid solution treatment temperature, then increases and reaches a trough at 1000 °C. This phenomenon can be attributed to the generation of a substantial amount of residual austenite in the 20CrNiMo at 1000 °C, leading to a reduction in hardness at this juncture.

**Figure 8:**
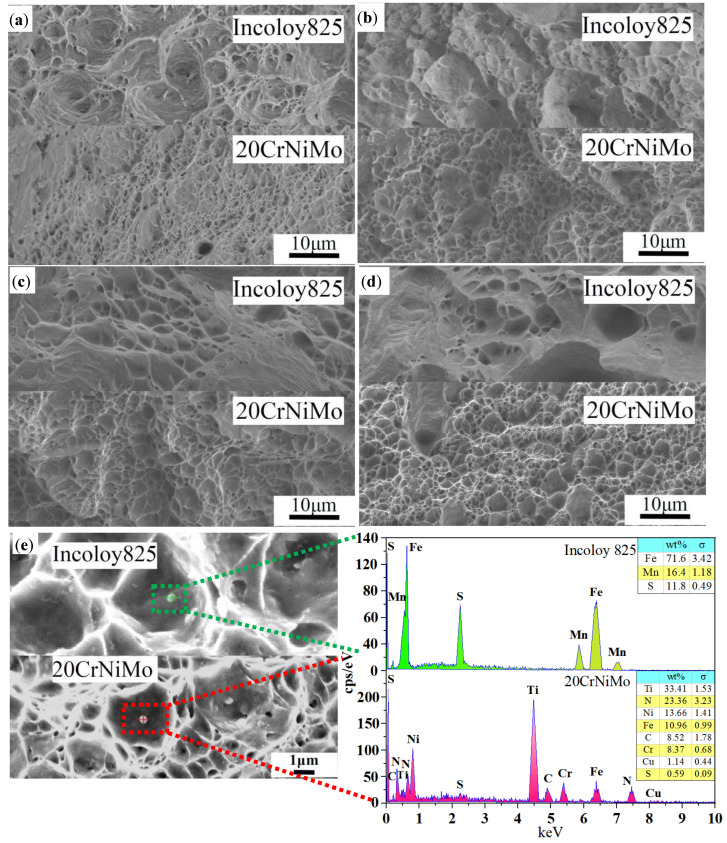
The interfacial shear stress–displacement curves and shear strength of 20CrNiMo/Incoloy 825-clad plates with different solid solution treatment processes: (**a**) 950 °C/1 h; (**b**) 1000 °C/1 h; (**c**) 1050 °C/1 h; (**d**) 1100 °C/1 h; (**e**) EDS analysis at 1050 °C/1 h.

The tensile fracture surfaces were scanned and analyzed in order to further analyze the cause of the fracture of the tensile specimens. Figure 8 shows that the fracture of both the Incoloy 825 layer and the 20CrNiMo layer after solid solution treatment exhibited dimple fractures. Additionally, a small amount of the second phase was detected at the bottom of the dimples, which was analyzed using energy-dispersive spectroscopy. The analysis indicated that the second phase on the 20CrNiMo was likely to be Ti(CN), while the second phase on the Incoloy 825 was likely to be MnS. During the tensile process, 20CrNiMo steel fractured before Incoloy 825. The primary reason for this phenomenon is that 20CrNiMo is an alloy structural steel with a yield strength typically between 785 and 980 MPa. In contrast, Incoloy 825 is a nickel–iron–chromium alloy known for its excellent corrosion resistance and relatively high strength, with a yield strength generally around 205 to 310 MPa. 20CrNiMo is more likely to reach its yield strength compared to Incoloy 825 when subjected to the same external forces, such as stretching or bending. As the external force gradually increases, 20CrNiMo undergoes plastic deformation first. Due to its lower strength limit, it reaches the material’s fracture strength and subsequently fractures under continued loading conditions. Additionally, the toughness of 20CrNiMo is generally lower than that of Incoloy 825. When small cracks occur in 20CrNiMo, they are more likely to propagate, leading to material fracture. This indicates that the fracture of the 20CrNiMo/Incoloy 825 composite material is mainly due to the presence of the hard and brittle second phase Ti(CN) in the20CrNiMo steel. These second phases disrupt the connectivity between the microstructures of the steel, causing stress concentration and the formation of micro-voids during the strong slip process at the grain boundaries. These micro-voids accumulate and grow into microcracks, which continue to expand, ultimately leading to the fracture of the steel.

### 3.3. Effect of Solid Solution Treatment on Tensile Shear Properties

**Figure 9:**
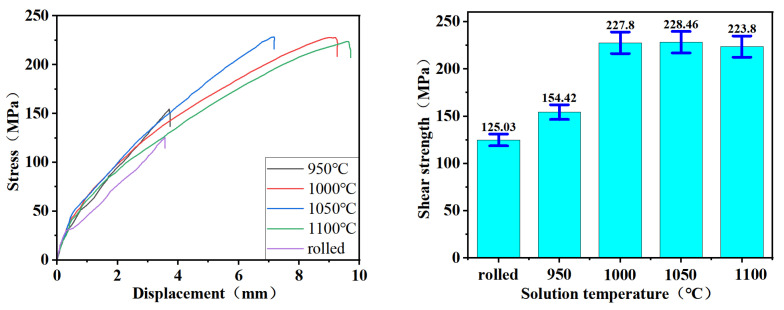
The shear stress–displacement curves and shear strength of 20CrNiMo/Incoloy 825 composite materials with different solid solution treatment processes.

Tensile shear experiments were carried out on 20CrNiMo/Incoloy 825 composite materials both before and after undergoing a solid solution treatment. The resulting shear stress–displacement curves and shear strengths are presented in Figure 9. It is evident from Figure 9 that all the shear stress–displacement curves displayed three distinct stages of deformation: elastic deformation, plastic deformation, and fracture [25]. Post-rolling, the shear strength of the 20CrNiMo/Incoloy 825 composite materials was 125.03 MPa. The interfacial bonding strength of the composite materials displayed improvement following solid solution treatment at varying temperatures. Specifically, at a solid solution temperature of 950 °C, the shear strength was elevated to 154.42 MPa. Subsequently, with a further increase in the solid solution treatment temperature, the shear strength increased again and then tended to be stable, and the highest shear strength reached 228.46 MPa when the solution temperature was 1050 °C. As per the stipulations of standard GB T 8165–2008, the minimum required interfacial shear strength is τ ≥ 210 MPa for class I/II composite steel plates and τ ≥ 200 MPa for class III composite steel plates. The shear strength of the composite material was relatively high when the solid solution temperature was 1050 °C and 1100 °C, which is in line with the strength standards of stainless steel composite materials.

**Figure 10:**
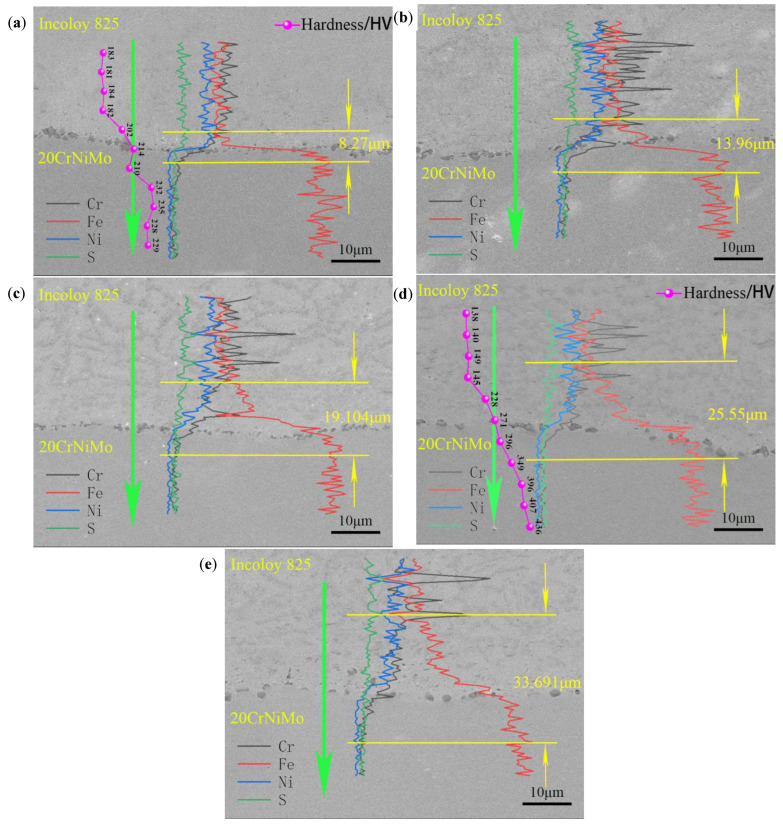
EDS line scan at the interface of 20CrNiMo/Incoloy 825 composite material: (**a**) EDS line scan before solid solution treatment; (**b**) 950 °C; (**c**) 1000 °C; (**d**) 1050 °C; (**e**) 1100 °C.

Line scanning was carried out on 20CrNiMo/Incoloy 825 composite materials both before and after undergoing solid solution treatment in order to better examine the effect of solid solution treatment on the interfacial bonding of 20CrNiMo/Incoloy 825 composite materials, as shown in Figure 10. In the figure, the upper layer of the interface is Incoloy 825, and the lower layer of the interface is 20CrNiMo.

Figure 10 shows the diffusion of elements at the interface of 20CrNiMo/Incoloy 825 composite materials before and after solid solution treatment. As can be seen in Figure 10, before solid solution treatment, the gradient of change in Cr, Ni, and Fe elements at the interface of 20CrNiMo/Incoloy 825 composite materials was steep, with a diffusion distance of approximately 8.27 μm, indicating that the diffusion of elements occurred in 20CrNiMo/Incoloy 825 composites before the solid solution treatment. Subsequent to solid solution treatment, the diffusion distance of elements at the interface of the composite materials increased from 13.96 um to 33.691 um with the increase in solid solution treatment temperature. This suggests that a large amount of elemental diffusion and migration occurred at the interface of 20CrNiMo/Incoloy 825 composites after solid solution treatment, contributing to the enhancement of interfacial bonding properties. The main reason for the generation of trace element diffusion was that the higher solid solution treatment temperature induced the full diffusion of Cr, Ni, and Fe atoms at the interface and the softening of the composite material, which was indispensable for increasing the thickness of the diffusion layer and toughening the composite interface. Consequently, the shear strength of 20CrNiMo/Incoloy 825 composites was significantly enhanced after solution treatment at 1050 °C/1 h and 1100 °C/1 h. Nonetheless, the higher temperature triggered the broadening of the decarburization layer of the base material and the increase in the number of brittle phases at the interface of the composite, diminishing the uniform plastic deformation capacity of the composite material during the tensile process [23]. Therefore, the elongation at 1050 °C and 1100 °C exhibited a reduction, as shown in Figure 7c.

The microhardness of the specimens before and after solid solution treatment was tested perpendicular to the composite interface, as shown in Figure 10a,d. The results show that the hardness of 20CrNiMo before solid solution treatment was about HV235 (100 g). After solid solution treatment at 1050 °C/1 h, the hardness of 20CrNiMo was about HV400 (100 g), which can be attributed to the formation of a martensitic microstructure induced by the oil quenching method employed during the solid solution treatment, resulting in an elevation of hardness. After solid solution treatment, the hardness of Incoloy 825 was lower than that before solid solution treatment, which was due to the softening of the structure caused by recrystallization and recovery, resulting in reduced hardness. As can be seen in Figure 10a,d, the hardness on the 20CrNiMo side proximate to the interface exhibited values of HV210 (100 g) and HV296 (100 g) before and after solid solution treatment, respectively, both lower than the matrix hardness of 20CrNiMo. This phenomenon can be linked to the decarburization behavior of 20CrNiMo steel, which alters the microstructure at the interface, consequently reducing hardness. On the other hand, the microhardness on the Incoloy 825 side close to the interface registered values of HV202 (100 g) and HV228 (100 g) before and after solid solution treatment, respectively, significantly surpassing the matrix hardness of Incoloy 825. This can be attributed to the diffusion of Fe and Cr, facilitating the precipitation of hard and brittle FeCr within the transition layer on the Incoloy 825 side, thereby elevating the composite material’s hardness [14]. Furthermore, KAM diagrams revealed a higher dislocation density in Incoloy 825 near the interface. These dislocations impede dislocation movement, resulting in a heightened microhardness on the Incoloy 825 side near the interface, causing the hardness of the Incoloy 825 side to be higher than that of the Incoloy 825 matrix.

## 4. Discussion

### 4.1. Selection of Parameters for the Solid Solution Treatment Process

The effect of the solid solution treatment on the interfacial structure indicates that the treatment temperature has a more significant impact on the decarburization and carburization layers at the interface than the treatment time. Thus, the influence of the solid solution treatment temperature on the structure and properties of 20CrNiMo/Incoloy 825 composite materials was specifically investigated in this study.

Ding Yun et al. [15] observed that when the Cr element diffused from Incoloy 825 to 20CrNiMo steel, a substantial amount of Cr_23_C_6_ precipitated along the grain boundary of the transition layer on the 20CrNiMo side. They found that longer heat preservation times resulted in more Cr_23_C_6_ precipitation [16]. Chenjun Yu et al. [26] found that the presence of Cr_23_C_6_ was detrimental to the composite materials, making them more susceptible to intergranular fracture, and it was necessary to shorten the solid solution treatment holding time. Additionally, it was found that a large number of M_23_C_6_ precipitation phases appeared in Incoloy 825 at solid solution treatment temperatures of 980 °C to 1015 °C, which led to a decrease in the corrosion resistance of Incoloy 825 [27]. To ensure the corrosion resistance of Incoloy 825 and prevent the formation of Cr_23_C_6_ along the grain boundaries of the 20CrNiMo side, high-temperature and short-term solid solution treatment parameters of 950 °C to 1100 °C for 1 h were chosen.

It can be seen from the effect of solid solution treatment on the interface microstructure that when the cooling mode was air cooling, the cooling rate was slow, and the final microstructure was ferrite and a small amount of granular pearlitic microstructure; when the cooling mode was oil cooling, the cooling rate was fast, and the final microstructure was martensite. The oil cooling solid solution treatment process was selected in order to ensure the hardness of the base layer of the composite material after solid solution treatment.

### 4.2. Determination of the Solid Solution Treatment Process

The effect of the solid solution treatment on the tensile strength demonstrates that, with the joint effects of dislocation strengthening [28] and solid solution strengthening [29], the tensile strength increased with the rise in solid solution treatment temperature. Notably, the highest tensile strength of 956.007 MPa was achieved at a solid solution treatment temperature of 1050 °C.

Based on the effect of solid solution treatment on hardness, it is evident that Incoloy 825 exhibited a trend of decreasing hardness as the solid solution treatment temperature increased. Conversely, the hardness of 20CrNiMo initially decreased and subsequently increased with the rise in solid solution treatment temperature, reaching its minimum at 1000 °C. At solid solution treatment temperatures ranging from 980 °C to 1015 °C, the emergence of a large number of face-centered cubic M_23_C_6_ precipitation phases and intermetallic compounds rich in Cr, Ni, Fe, and Mo occurred within Incoloy 825, leading to increased grain boundary corrosion [30,31]. Upon reaching 1050 °C, these precipitated phases dissolved, consequently enhancing Incoloy 825’s corrosion resistance. Solid solution treatment temperatures of 1050 °C and 1100 °C were selected to ensure the retention of good strength and hardness in the 20CrNiMo substrate and to maintain the corrosion resistance of the Incoloy 825 cladding.

According to the effect of solid solution treatment on the interfacial bonding properties, it can be seen that the higher solid solution treatment temperature induced the full diffusion of Cr, Ni, and Fe atoms at the interface and the softening of the matrix, which played an essential role in the increase in the thickness of the diffusion layer and the toughening of the composite interface [32]. Because of the increased bonding force at the composite interface, the shear strength increased with the solid solution treatment temperature. The highest shear strength of 228.46 MPa was obtained at the solid solution treatment temperature of 1050 °C.

**Figure 11:**
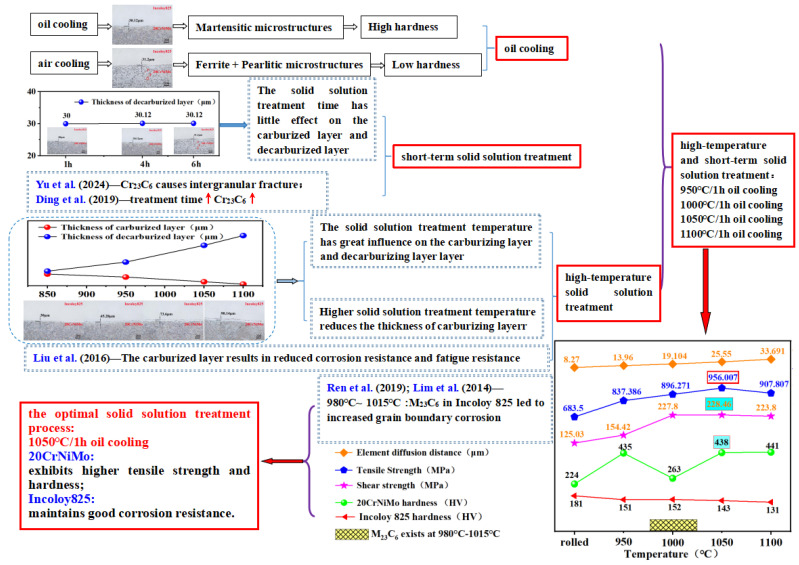
Experimental conclusion diagram. Ding [15]; Liu et al. [17]; Yu et al. [26]; Ren et al. [27]; Lim et al. [30].

In summary, the 20CrNiMo/Incoloy 825 composite materials exhibited optimal interfacial bonding properties after the 1050 °C/1 h oil cooling solid solution treatment. Simultaneously, the 20CrNiMo substrate demonstrated high tensile strength and hardness, while the Incoloy 825 cladding maintained good corrosion resistance, collectively establishing excellent comprehensive mechanical properties for the 20CrNiMo/Incoloy 825 composite materials. Therefore, the optimal solid solution treatment process for 20CrNiMo/Incoloy 825 composite materials is the 1050 °C/1 h oil cooling treatment. The experimental conclusions are shown in Figure 11.

Although the optimal solution treatment temperature for 20CrNiMo/Incoloy 825 composite materials was identified in this study, there are still some deficiencies in this article. When using the MATLAB software to process EBSD data, it is currently only possible to analyze images of a single material, which means that both 20CrNiMo steel and Incoloy 825 could not be processed simultaneously. This limitation impacted the accuracy of the MATLAB data. It is hoped that further progress can be made in this area in the future.

## 5. Conclusions

(1) It was observed from the effect of solid solution treatment on the microstructure that the solid solution treatment temperature had a greater effect on the interface decarburization layer and carburization layer than the solid solution treatment time. The thickness of the carburization layer decreased, while the thickness of the decarburization layer increased with the rise in solid solution treatment temperature. The original austenite grain size of 20CrNiMo and Incoloy 825 increased as the solid solution treatment temperature rose, while the residual austenite content first increased and then decreased at higher temperatures.

(2) According to the analysis of the tensile properties, microhardness, and fracture morphology following solid solution treatment, the tensile strength and elongation exhibited an initial increase with increasing solid solution treatment temperature, followed by a decrease. A toughness fracture occurred in 20CrNiMo/Incoloy 825 composite materials, and the reason for the fracture was mainly the stress concentration caused by the second phase of Ti(CN), which led to the formation of microcracks inside the composite material and ultimately resulted in the fracture of the steel. With an increase in the solid solution treatment temperature, the hardness of Incoloy 825 showed a decreasing trend, while the hardness of 20CrNiMo first decreased and then increased.

(3) The analysis of the effect of the solid solution treatment on the shear performance and interface element diffusion revealed that the higher solid solution treatment temperature induced the complete diffusion of Cr, Ni, and Fe atoms at the interface and the softening of the matrix. This played an important role in increasing the thickness of the diffusion layer and toughening the composite interface, facilitating an enhanced interfacial bonding force and subsequently increasing the shear strength.

(4) 20CrNiMo/Incoloy 825 composite materials underwent a 1050 °C/1 h oil cooling solution treatment, during which a large number of elements diffused and migrated at the interface, improving the interfacial bonding properties. At that time, the 20CrNiMo substrate had a high tensile strength and hardness, while the Incoloy 825 cladding exhibited good resistance to corrosion, indicating that it had excellent comprehensive mechanical properties. Therefore, the optimal solid solution treatment process for 20CrNiMo/Incoloy 825 composite materials is 1050 °C/1 h oil cooling.

## Figures and Tables

**Table 1:** Chemical composition of Incoloy 825 and 20CrNiMo steel (wt%).

**Incoloy 825**	C	Cr	Ni	Mo	Mn	Si	S	P	Fe	
	0.19	0.54	0.51	0.25	0.72	0.28	0.005	0.012	Bal.	
**20CrNiMo**	C	Cr	Ni	Mo	Mn	Si	Ti	Al	Cu	Fe
	0.016	21.46	39.35	2.84	0.48	0.37	0.97	0.11	2.05	Bal.

## Data Availability

The original contributions presented in the study are included in the article, further inquiries can be directed to the corresponding author/s.
